# Hybrid Coatings Based on Polyvinylpyrrolidone/Polyethylene Glycol Enriched with Collagen and Hydroxyapatite: Incubation Studies and Evaluation of Mechanical and Physiochemical Properties

**DOI:** 10.3390/jfb15030062

**Published:** 2024-03-01

**Authors:** Dagmara Słota, Josef Jampilek, Agnieszka Sobczak-Kupiec

**Affiliations:** 1 Department of Materials Science, Faculty of Materials Engineering and Physics, Cracow University of Technology, 37 Jana Pawła II Av., 31-864 Krakow, Poland; agnieszka.sobczak-kupiec@pk.edu.pl; 2 Department of Analytical Chemistry, Faculty of Natural Sciences, Comenius University, Ilkovicova 6, 842 15 Bratislava, Slovakia; 3 Department of Chemical Biology, Faculty of Science, Palacky University, Slechtitelu 27, 783 71 Olomouc, Czech Republic

**Keywords:** coatings, ceramic, polymer, hydroxyapatite, collagen, polyvinylpyrrolidone, polyethylene glycol, glutathione

## Abstract

Coating materials offers an intriguing solution for imparting inert implants with additional bioactive characteristics without changing underlying parameters such as mechanical strength. Metallic implants like endoprostheses or polymeric implants can be coated with a thin layer of bioactive film capable of stimulating bone-forming cells to proliferate or release a drug. However, irrespective of the final implantation site of such a coating biomaterial, it is necessary to conduct detailed mechanical and physicochemical in vitro analyses to determine its likely behavior under biological conditions. In this study, polymeric and composite coatings with hydroxyapatite obtained under UV light underwent incubation tests in four different artificial biological fluids: simulated body fluid (SBF), artificial saliva, Ringer’s fluid, and water (as the reference fluid). The potentiometric and conductometric properties, sorption capacity, and degradation rate of the coatings were examined. Furthermore, their hardness, modulus of elasticity, and deformation were determined. It was demonstrated that the coatings remained stable in SBF liquid at a pH value of around 7.4. In artificial saliva, the greatest degradation of the polymer matrix (ranging between 36.19% and 39.79%) and chipping of hydroxyapatite in the composite coatings were observed. Additionally, the effect of ceramics on sorption capacity was determined, with lower capacity noted with higher HA additions. Moreover, the evaluation of surface morphology supported by elemental microanalysis confirmed the appearance of new apatite layers on the surface as a result of incubation in SBF. Ceramics also influenced mechanical aspects, increasing hardness and modulus of elasticity. For the polymer coatings, the value was 11.48 ± 0.61, while for the composite coating with 15% ceramics, it increased more than eightfold to a value of 93.31 ± 11.18 N/mm^2^. Based on the conducted studies, the effect of ceramics on the physicochemical as well as mechanical properties of the materials was determined, and their behavior in various biological fluids was evaluated. However, further studies, especially cytotoxicity analyses, are required to determine the potential use of the coatings as biomaterials.

## 1. Introduction

The coating technique consists of covering the surface of an implant with a layer of another biomaterial [1]. Coating modifies the surface of biomaterials and the biological response of the host tissue in the peri-implant area [2]. In the case of bone implants, bioactive ceramics, such as hydroxyapatite (HA), are of particular interest in this aspect; they spontaneously form a bony apatite layer on their surface in a living organism and fuse with bone through this layer. These types of materials are of great clinical importance as bone-restorative materials. The formed apatite is highly similar to bone mineral in its composition and structure. Therefore, osteoblasts preferentially proliferate and differentiate to produce apatite as well as collagen on this layer of apatite. When an inert material (whether polymer or metallic) is coated with hydroxyapatite, bone cells adhere to the surface of the apatite coating without intermediate layers. Later, the hydroxyapatite matrix of bone cells becomes integrated with the coating, resulting in excellent adhesion of the coated implant to the bone [3,4,5].

Many biologically active compounds can be used to manufacture coatings, thereby providing some additional biological functions [6,7]. Collagen is a polymer of natural origin that has been used in medicine and cosmetology for more than 50 years. Due to its wide range of properties, it is used as a material or intermediate in wound healing dressings, dentistry, scaffold design for osteochondral engineering, otolaryngology, and aesthetic medicine [8,9]. Otolaryngology is a medical field focusing on the ears, nose, and throat. It also includes head and neck surgery [10]. Collagen is the most abundant protein in the extracellular matrix in mammals. It is a non-toxic biopolymer that is highly biodegradable. Moreover, it demonstrates poor immunogenicity and very good degradability. Currently, about 29 types of collagen proteins are known, which are characterized by their diverse and unique structures [11]. Depending on the structure and function, many types are distinguished. The most important are collagen types I and II. The first one constitutes about 70% of all collagen types, and is mainly found in ligaments, bones, and tendons. The second is the main component of vitreous cartilage and the main collagen of the vitreous body, as well as the nucleus pulposus of intervertebral discs. The presence of collagen ensures flexibility and improves tissue condition [12,13,14]. In biomedical and implant applications, fish collagen is most commonly used. It can take both fibrillar and non-fibrillar forms and is distinguished by lower melting and gelling temperatures than collagens extracted from mammals [15,16].

Glutathione (GSH) is an interesting biologically active tripeptide. It is a compound that demonstrates strong antioxidant properties, but beyond that, it is involved in many other functions [17]. It is responsible for recycling vitamins E and C, transporting amino acids, and producing coenzymes. Importantly, with age, GSH levels gradually decrease, so its external delivery is important [18,19,20].

Another biologically active ingredient that can significantly affect the acceleration of regeneration of the skeletal or cartilaginous system is the above-mentioned HA. It is a ceramic that occurs naturally in the body forming the mineral phase of bone (constituting about 70%, and the remaining 30% is mainly collagen proteins) [21,22]. Synthetic HA used in implantology can chemically bind to surrounding tissues; it has osteoconductive properties and the ability to stimulate bone-forming cells to proliferate.

Regardless of the type, due to a variety of processes occurring in the body, biomaterials must be subjected to detailed physicochemical or mechanical analysis. Different physiological fluids exhibit different chemical compositions or pH values, so they can interact differently with implanted foreign bodies. Artificial biological fluids are used to simulate the biological environment in an in vitro laboratory setting. The most common of these are simulated body fluid (SBF), artificial saliva, and Ringer’s fluid, whose composition corresponds to extracellular fluid [23,24].

The study of the interaction of materials with fluids allows the monitoring of a range of parameters such as ionic conductivity, changes in pH values, degree of degradation, and swelling coefficient. They can vary depending on the composition of biomaterials. Potentiometric and conductometric tests help determine whether hazardous components are released or precipitate from the interior of the material during fluid interactions, which could negatively affect the cellular balance. Any spike in pH values to highly alkaline or acidic is a cause for concern, since cells in the implant area are unable to proliferate under such conditions [25,26]. Degradation studies provide information on how quickly a biomaterial will disintegrate into smaller molecules or fragments and can be replaced by newly growing tissue. However, both degradation and swelling capacity are also strategies for delivering active substances including drugs in targeted therapies. As a result of gradual degradation, active substances that are physically or chemically bound to the material are released. In the case of swelling, drug delivery involves the gradual elution from polymer chains following the penetration of the liquid medium deep into the network [27,28,29]. Given the above, in vitro studies are recommended before proceeding to cellular or in vivo studies on animal models.

**Figure 1:**
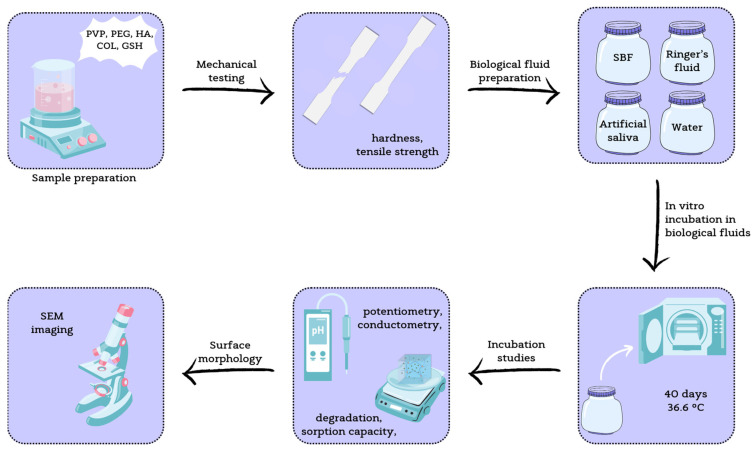
Workflow of the study, from sample preparation to individual analysis.

The work presented in this paper continues the study of innovative composite coatings containing polyvinylpyrrolidone (PVP), polyethylene glycol (PEG), eGSH, COL, and bioactive HA for bone tissue regeneration [30,31]. The composite coating guarantees improved properties because it combines the features of polymeric and ceramic materials. Ceramics are characterized by brittleness, so they are unable to carry loads; however, suspending them in a polymeric hydrogel matrix can overcome this problem without losing their bioactive character. The aim of the study was to evaluate the mechanical and physicochemical properties of the developed materials and to determine their potential as active substance carriers by determining the sorption capacity in selected simulated biological fluids. It should be emphasized that the developed materials have great potential due to the high biological value of the components used in their synthesis, such as glutathione and hydroxyapatite, which promote osteogenesis. No other solution of this type has been found so far. Figure 1 presents the research methodology of the study.

## 2. Materials and Methods

### 2.1. Reagents

The reagents used for the synthesis of HA, i.e., calcium acetate monohydrate (Ca(CH_3_CO_2_)_2_·H_2_O), sodium phosphate dibasic (Na_2_HPO_4_), and ammonia water (NH_4_OH, 25%), as well as polymers, i.e., polyethylene glycol (PEG), polyvinylpyrrolidone (PVP), poly(ethylene glycol) diacrylate Mn 575 (PEGDA), collagen from bovine Achilles tendon (COL), and other reagents, such as 2-hydroxy-2-methylpropiophenone 97% and peptide l-glutathione reduced 98% (GSH), were obtained from Sigma-Aldrich (Darmstadt, Germany). For the preparation of Ringer’s solution and simulated body fluid (SBF) fluid, NaCl, KCl, CaCl_2_, and Na_2_SO_4_ from Eurochem BGD (Tarów, Poland) were used. Additionally, NaHCO_3_ from DOR-CHEM (Krakow, Poland), K_2_HPO_2_·3H_2_O and MgCl_2_·6H_2_O from Chempur (Piekary Slaskie, Poland), 2-amino-2-(hydroxymethyl)- propane-1,3-diol (Tris) from POCH (Gliwice, Poland), and HCl 35–38% solution from Stanlab (Lublin, Poland) were purchased.

### 2.2. Preparation of Coatings

The coatings were prepared as previously described [30], and their composition is presented in Table 1. The crosslinking process was used, exposing the samples to UV light for 4 min with a lamp power of 0.8 J/cm^2^, and a distance from the lamp of 5 cm.

**Figure 2:**
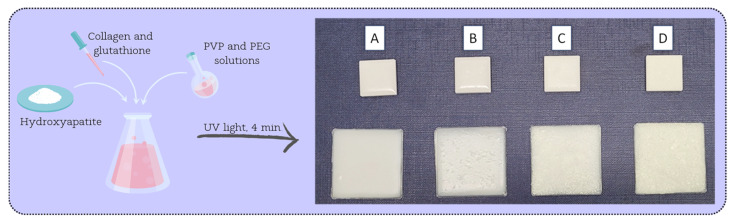
Coatings applied to polymer plates. Smaller squares above—PLA plates obtained by 3D printing. Larger squares below—PLA plates obtained by injection molding.

The developed method of coating preparation and application is a completely waste-free technique and generates no by-products. Ultraviolet light crosslinking resulted in a fully crosslinked material, and no crumbling of the ceramics was observed even at higher HA concentrations. Also, good integrity was obtained as the coatings were fully continuous and uniformly crosslinked with no significant irregularities or holes. Figure 2 demonstrates the resulting materials applied to hard polylactide (PLA) plates.

### 2.3. Methodology of Incubation Tests

#### 2.3.1. Fluid Preparation

A pH-metric study of the coatings was conducted to determine their bioactivity. Three incubation fluids were selected: SBF (simulated body fluid), artificial saliva, and Ringer’s solution (60 mL). These were placed in sterile sealed containers, and 1 g coating discs were immersed in them.

The purpose of this study was to confirm the interactions occurring between the sample and the incubation fluids. The molecules and ions contained in the fluids, interacting with the biomaterial, cause a change in pH value. The materials were incubated in a POL-EKO incubator, model ST 5 B SMART (Wodzisław Śląski, Poland), at 36.6 °C for 40 days. The pH values were measured using an Elmetron CX-701 multifunctional device (Zabrze, Poland).

Ringer’s fluid and SBF were prepared according to the details in Table 2, Table 3 and Table 4. For all fluids, each subsequent component was added after the previous component was completely dissolved. Ringer’s solution was obtained at room temperature. To prepare the SBF solution, 700 mL of distilled water was heated to 36.5 °C (±0.5 °C). One by one, all the ingredients were added, and finally, with a solution of HCl and (CH_2_OH)_3_CNH_2_, the pH was brought to 7.4–7.45. After that distilled water was added to a volume of 1000 mL.

#### 2.3.2. Electrochemical Analysis—Potentiometry

Potentiometric analysis was carried out in order to determine the change in pH values of the solutions in which the coatings were incubated. This assay enables the in vitro stability of the material to be determined in an incubation medium, which simulates conditions in a living organism by its composition. The purpose of this research was to confirm the interactions occurring between the sample and the incubation medium. The ions and molecules contained in the fluids, interfering with the biomaterial, cause a change in the pH value. The obtained dried coating samples with an initial mass of 1 g were incubated at a constant temperature of 36.6 °C in the POL-EKO incubator, model ST 5 B SMART, in prepared artificial biological fluids and distilled water (100 mL) for 40 days. Distilled water was chosen as the reference fluid. Three replicates were performed for each coating composition. The pH value of the fluids was systematically measured using the Elmetron CX-701 multifunctional device with an EPS-1 pH-metric electrode (Zabrze, Poland).

#### 2.3.3. Electroanalytical Analysis—Conductivity

Conductometric analysis was carried out in order to evaluate the ionic conductivity in the incubation medium. The ion exchange occurring at the material/liquid interface causes changes in the conductometric value, as well as gives an indication that the material is not inert under the given conditions. The movement of ions and the appearance of their increasing number causes changes in the conductivity value. Analogous to potentiometric measurement, dried samples of 1 g were placed in artificial biological fluids and distilled water as the reference (100 mL) and incubated in a POL-EKO incubator, model ST 5 B SMART, for 40 days at a constant temperature of 36.6 °C. Three replicates were performed for each coating composition. The conductometric value of the fluids was systematically measured using the Elmetron CX-701 multifunctional device with an ECF-1 conductivity sensor (Zabrze, Poland).

#### 2.3.4. Determination of Sorption Capacity

One of the strategies for delivering drugs and others active ingredients in targeted therapies is to determine the swelling capacity of materials. The composition of composite materials can affect not only their structure, but also the ability of these materials to swell (to bind water within their structure). In order to investigate the relationship between composition and swelling ability, a swelling kinetics study was carried out. For this purpose, 1 g discs were placed in sterile containers filled with the selected incubation fluids, i.e., SBF, artificial saliva, Ringer’s fluid, and distilled water as the reference (100 mL), at 36.6 °C. After 15 min, the samples were removed, excess liquid was collected using filter paper, and the samples were weighed. The measurement was repeated for all samples analogously after 15 min, 30 min, 45 min, 1 h, 2 h, 24 h, 7 days, and 14 days. Three replicates were performed for each coating composition.

The swelling ability of the coatings immersed in fluids was calculated in accordance with the following formula (Equation (1)):

(1) $$\mathrm{Swelling}\;\mathrm{ability}=\frac{m_{1}-m_{0}}{m_{0}}\cdot 100\%$$

where *m*_1_ is the weight of the immersed sample (g) and *m*_0_ is the initial weight of the sample (g).

The kinetics of coating swelling was investigated using the Voigt-based viscoelastic model (Equation (2)) [32].

(2) $$S_{t}=S_{e}[1-e^{-\frac{t}{\tau }}]$$

where *S_t_* is swelling at a given time *t* (%), *S_e_* is equilibrium swelling (power parameter) (%), *t* is time of swelling *S_t_* (min), and *τ* is the rate parameter (min).

#### 2.3.5. Degradation Studies

Depending on the purpose of the biomaterial in the body as well as the functions they are expected to take over, it is expected that they will be stable or able to degrade over time. Degradation allows biomaterials to overgrow with new, natural tissue. To determine the stability of coatings in a biological environment, a degradation study was conducted. For this purpose, discs weighing approximately 1 g were placed in sterile containers filled with selected incubation fluids, i.e., SBF, artificial saliva, Ringer’s fluid (100 mL), and distilled water as the reference, at 36.6 °C. The containers remained unopened for a period of 60 days. Three replicates were performed for each coating composition. After this time, the samples were removed, dried, and weighed. Based on the equation below (Equation (3)), the degree of degradation in each liquid was determined.

(3) $$Degree\;of\;degradation=\frac{m_{t}-m_{t0}}{m_{t0}}\cdot 100\%$$

where *m*_t_ is the weight of the dry sample after incubation time (g) and *m*_t0_ is the initial dry sample mass at time t_0_ (g).

### 2.4. Surface Morphology

The imaging and observation of the structure of the materials provides information on the possible mineralization processes that may occur on the surface under the influence of interaction with the incubation medium. After a 14-day incubation in SBF, the coatings were dried and referred for examination. To determine differences in surface morphology before and after incubation in artificial biological fluids, imaging was performed using a JEOL 5510LV (Tokyo, Japan) scanning electron microscope (SEM) with an EDS IXRF detector. Before SEM measurement, the samples were lyophilized and coated with a conductive gold nanolayer. EDS microanalysis was performed with points in order to detect specific elements on the surface of the samples.

### 2.5. Hardness Measurement

The composition of the materials as well as the addition of ceramics can affect the mechanical parameters of the coatings obtained. In order to determine the influence of the ceramic phase on the hardness of the coatings, Shore A hardness was measured using a ZwickRoell 3130 (Ulm, Germany). The measurement was performed at a load of 10 N. The instrument was pressed against the material. The indenter, extending from the base, was pressed into the material, as a result of which the balance between the force of the spring and the response of the material was established. Once the equilibrium is established, the pointer stops at the corresponding range of the scale represented in Shore degrees (0–100) [33]. Three replicates were performed for each coating composition. The hardness of the materials was measured by the Shore method according to the PN-ISO 868 standard, with an indenter according to the PN-93/C-04206 standard [34,35].

### 2.6. Static Tensile Test

Biomaterials in the environment of a living organism are subjected to various loads; hence, it is crucial to study not only their physicochemical but also mechanical parameters. Static tensile tests were carried out on a Shimadzu AGS-X 10 kN testing machine (Kyoto, Japan). The tensile test was conducted in accordance with the PN-EN ISO 527-1 standard at a crosshead speed of 1 mm/min [36]. A load value of 5 N was applied. Longitudinal paddle-shaped coating samples were prepared in order to perform the static tensile test. Three replicates were performed for each coating composition.

### 2.7. Statistical Analysis

The results of the experiments were subjected to statistical analysis. Statistical significance was calculated using one-way analysis of variance (ANOVA) (alpha value = 5%). The sorption capacity of the coatings and hardness measurement results were subjected to this analysis. For all other experiments, measurements were performed in triplicate and are presented as mean value and standard deviation (SD).

## 3. Results

### 3.1. Results of Incubation Tests

#### 3.1.1. Electrochemical Analysis—Potentiometry

**Figure 3:**
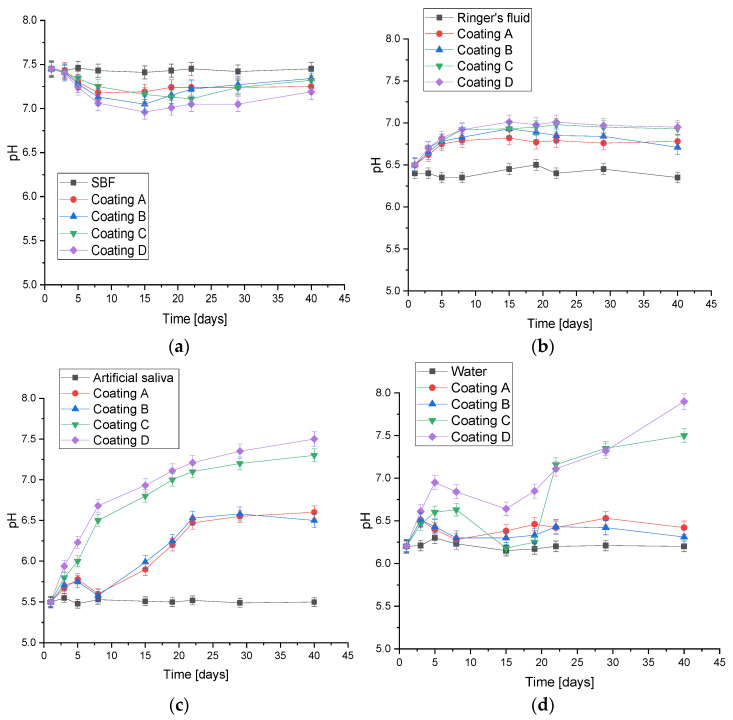
Potentiometric analysis of coatings during 40-day incubation in: (**a**) SBF; (**b**) Ringer’s fluid; (**c**) artificial saliva; (**d**) distilled water (number of repetitions *n* = 3).

The greatest changes in pH value (Figure 3) were observed in artificial saliva, where the pH value increased from 5.5 all the way up to a value of 7.5 for ceramic coatings (**C** and **D**) and around 6.5 for polymer coatings (**A** and **B**). During incubation in artificial saliva, small pieces of the coating were observed on the bottom of the vessel, which may suggest partial degradation of the material. The higher pH value for coatings **C** and **D** is likely due to the leaching of hydroxyapatite, which determines a higher, even more alkaline pH. In SBF, the materials exhibited the greatest stability, with the pH ranging between 7 and 7.5, meaning safety for the body. However, SBF itself has buffering properties, which probably explains the slight changes. The coatings behaved similarly in Ringer’s fluid, where an increase to a maximum value of approximately 7 was observed from the first day and the initial pH value of 6.5. This is good information, since physiological fluids in the body have a standard pH value between 7 and 7.5. Depending on diet, lifestyle, and health condition, it is about 7.35–7.45 for blood [37].

#### 3.1.2. Electroanalytical Analysis—Conductivity

**Figure 4:**
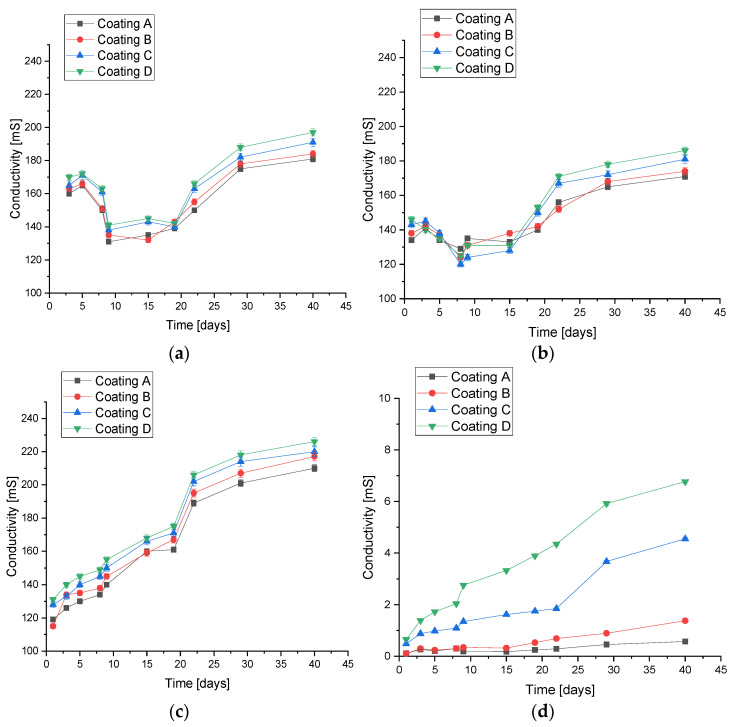
Conductivity analysis of coatings during 40-day incubation in (**a**) SBF; (**b**) Ringer’s fluid; (**c**) artificial saliva; and (**d**) distilled water (number of repetitions *n* = 3).

In parallel to measuring changes in pH values, the ionic conductivity of electrolytes (SBF, water, Ringer’s fluid, distilled water) was monitored over time (Figure 4). The value of conductivity depends on the amount of ions present in the solution during incubation and changes with their concentration. This confirms the interactions occurring between the coatings and the medium as well as ongoing ion exchange. The smallest differences in conductivity were observed for coatings in distilled water. Pure distilled water is practically devoid of any ions that could interact with coatings; however, due to the penetration of the fluid into the material, the leaching of under-crosslinked polymer solutions or ceramic grains probably occurred, resulting in small changes. It can be concluded that, for polymer coatings, practically no changes were recorded, as the difference in value was approximately 1 mS; for **C** and **D** coatings with hydroxyapatite, the differences from the initial values were about 4 and 7 mS, respectively. The largest substitutions were observed in artificial saliva, and were caused by the observed partial degradation. The conductivity value doubled from about 110 to about 220 on the last day of measurement. In the case of SBF and Ringer’s fluid, the samples behaved similarly, and the spikes in ionic conductivity values may indicate processes of continuous crystallization and recrystallization on the surface of the materials due to interaction with ions from the solutions.

#### 3.1.3. Determination of Sorption Capacity

**Figure 5:**
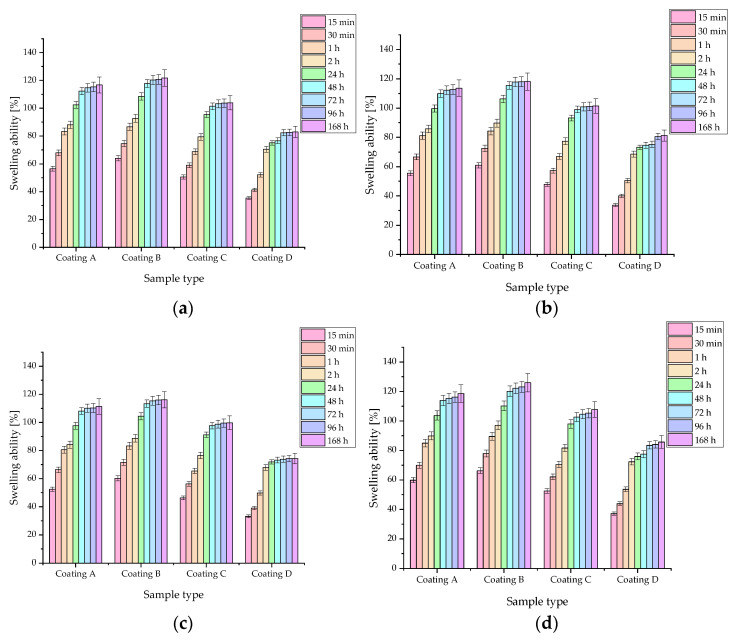
Sorption capacities determined by swelling ability (%) for samples in (**a**) SBF; (**b**) Ringer’s fluid; (**c**) artificial saliva; and (**d**) distilled water. According to statistical analysis: SBF *f*-ratio = 0.08937, *p* = 0.965651; Ringer’s fluid *f*-ratio = 0.2221, *p* = 0.880727; artificial saliva *f*-ratio = 0.39524, *p* = 0.756828; distilled water *f*-ratio = 0.45687, *p* = 0.7133633 (number of repetitions *n* = 3).

During incubation studies, swelling ability (Figure 5) and equilibrium swelling (Table 5) were determined for coatings in the four fluids: SBF, artificial saliva, Ringer’s fluid, and distilled water. An increase in swelling coefficients was observed for all samples over time. The results for distilled water have been presented previously; however, in this publication, they were chosen to be compared to other fluids [30]. It was observed that in all liquids, coating **B** exhibited the highest sorption capacity. In coatings **C** and **D**, hydroxyapatite was present, the grains of which limited the possibility of swelling by occupying the free spaces between the polymer chains. This resulted in smaller amounts of fluid being able to reach deeper into the material. On the other hand, coating **A** was lacking collagen fibers that were found in coating **B**. Collagen demonstrates the ability to bind large amounts of water molecules, which probably explains the highest observed sorption capacity [38]. The rate parameter (τ) was also determined, which, for all coatings, was the highest for water. Other liquids present a much richer chemical composition and contain more ions, and as a result, ions can react with each other and crystallize on the surface, or form additional crosslinks between polymer chains [39]. This results in an increase in the crosslinking density of the polymer matrix and consequently a reduction in the amount of free space where water molecules can reach, resulting in a reduction in sorption capacity.

For all fluids, the highest sorption capacity was observed during the first 48 h when changes were dynamic. After that time, the values slowly stabilized and varied little. However, it is important to highlight that swelling abilities were exhibited by all samples, potentially confirming their applicability as carriers of active substances.

#### 3.1.4. Degradation Studies

Degradation studies were conducted for 60 days in the four liquids. The conditions of a living organism were simulated. Each of the fluids had a slightly different initial pH value: for SBF, it was about 7.4, for Ringer’s fluid about 6.5, for artificial saliva about 5.5, and for distilled water about 6.5. The difference in weight before and after the incubation period was observed for all samples (see Table 6). The largest changes were observed for coatings in artificial saliva where the difference ranged from 36.19% for coating **A** to 39.79% for coating **D**. Significantly, in artificial saliva, a greater loss was observed for composite coatings, while in the other liquids, larger values were observed for polymer coatings **A** and **B**. This is probably caused by the low pH of saliva, which caused degradation of the polymer matrix, and eventually the ceramic began to crumble out of the coating as well. Fragments of coatings were observed on the bottom of the vessel during potentiometric measurements. The smallest changes were observed for SBF, where a value of only 8.14% was observed for coating **D**. These small differences are probably due to the continuous processes of crystallization and recrystallization on the surface of the coating due to the interaction of the biomaterials with the ions present in the liquid. It is likely that partial degradation occurs in SBF, as it occurs even in distilled water, but recrystallization processes make the loss percentages not so large.

### 3.2. Surface Morphology

**Figure 6:**
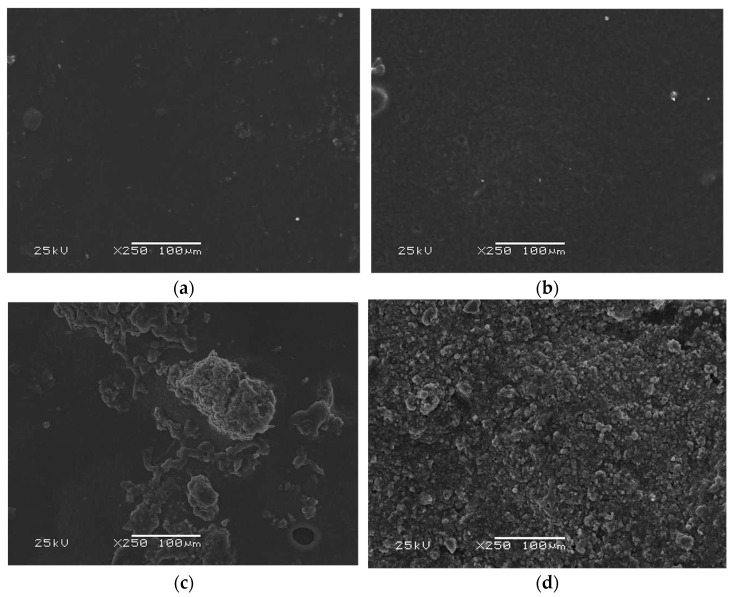
Morphology analysis of the coatings before the incubation period: (**a**) SEM image of coating **A**; (**b**) SEM image of coating **B**; (**c**) SEM image of coating **C**; (**d**) SEM image of coating **D**.

Figure 6 presents the surface morphology of the coatings at ×250 magnification before incubation in SBF.

Despite the presence of collagen in coating **B** and its absence in coating **A**, the surfaces of these two polymeric materials are similar and relatively smooth. Noticeably, as the proportion of HA in the materials increases, the number of crystals observed during measurement increases. In coating **C**, it is possible to indicate a polymeric phase as well as a ceramic phase, while in the case of coating **D**, the surface of the material is practically entirely covered with HA grains, with no polymeric free spots breaking through.

**Figure 7:**
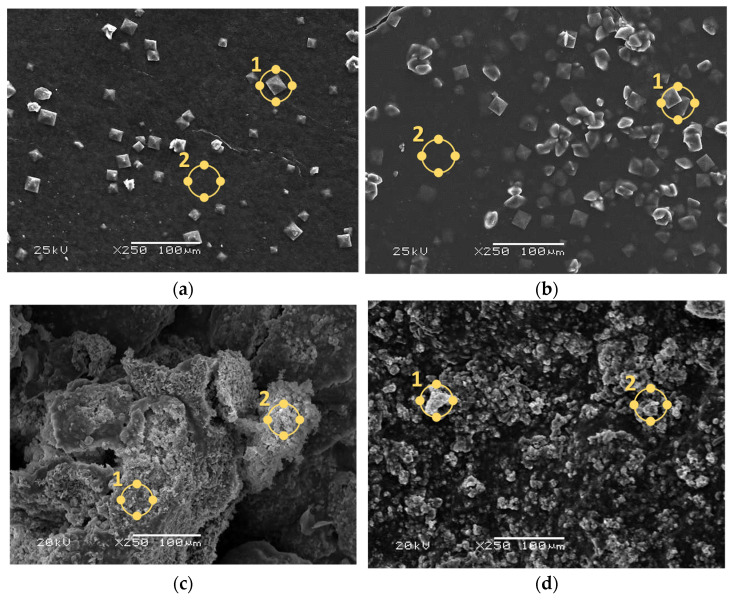
Morphology analysis of the coatings after the incubation period with EDS microanalysis points indicated by yellow circles: (**a**) SEM image of coating **A**; (**b**) SEM image of coating **B**; (**c**) SEM image of coating **C**; (**d**) SEM image of coating **D**.

The coatings were incubated in SBF liquid for a period of 14 days. Figure 7 demonstrates a microscopic image of the surface of the dried samples after this time. The precipitation of crystals with a characteristic square shape was observed on the polymer coatings. For coating **B**, there were slightly more of them, although they were still in comparable amounts. For coating **C**, comparing the image to that before the immersion period in SBF, the appearance of new crystals in the form of cauliflower-shaped efflorescence was observed, which covered the entire surface of the sample; no polymer phase was found penetrating to the surface. The **D** coating before and after the incubation period looks similar; however, after incubation, it appears to be rougher, which may be due to the precipitation of new apatite layers and crystals. The appearance of new apatite layers and crystals on the surface of incubated samples is a very desirable result. Such biomineralization is proof of the bioactivity of the coatings due to the evidence of interaction between the material and the fluid medium [39,40].

In parallel with the analysis of surface morphology by SEM, EDS microanalysis was carried out to determine the presence of individual elements. The elements C and O derived mainly from polymers, Ca and P derived from HA, and the composition of the SBF fluid, as well as Mg, Na, K, and Cl, whose ions were also found in SBF, were determined. Au, which was sputtered onto the samples before measurement, was not quantified. The results are presented in Table 7. For each coating, two spots on the surface were inspected. In composite coatings **C** and **D**, the compositions at the two sites tested are similar. High Ca and P contents indicate precipitated apatite layers. In the case of polymer coatings **A** and **B**, Ca ions were also observed on the surface, but the amount of P was disproportionately higher. Larger amounts of these elements are found in composite materials. It was also observed that there were more Cl ions on the surface of these coatings, which probably indicates the precipitation of sodium, potassium, or calcium chlorides from the solution on the surface. The detection of Mg, Na, and Cl after incubation indicates the desired reactions between the artificial saline fluid and the biomaterial.

Based on the obtained images and the amount of each element, it was demonstrated that the ability to form mineralized apatite layers on the surface of incubated materials is fully dependent on the presence of a ceramic phase in the coating. Therefore, a higher proportion of the ceramic phase in biomaterials is likely to increase their bioactivity through the ability to form new apatite layers. This phenomenon is related to the unique nature of SBF—more specifically, its ionic composition. Interactions between Ca^2+^ ions and negatively charged HA cause the spontaneous growth of apatite-like nuclei on the surface. In time, it is transformed into bone-like apatite through the incorporation of P ions [41].

### 3.3. Hardness Measurement

**Figure 8:**
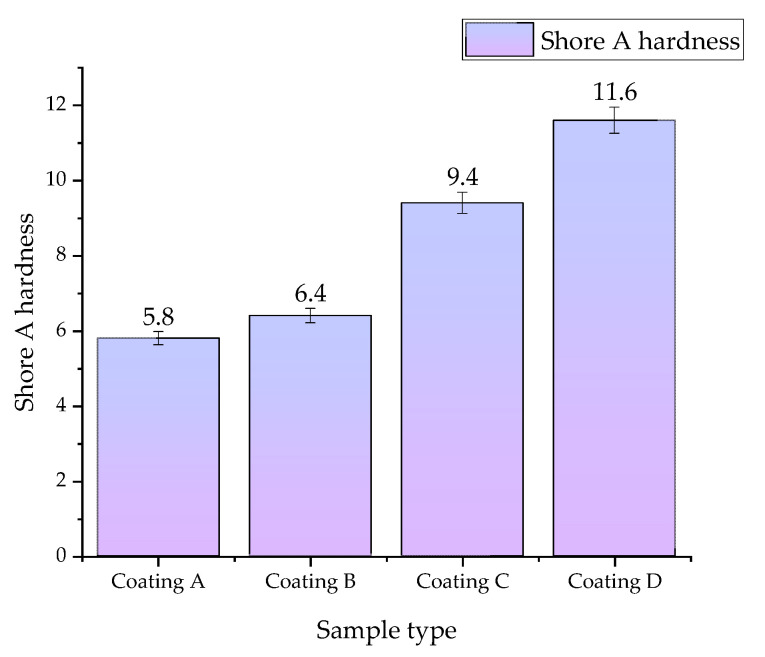
Hardness measurements of coatings performed by the Shore A method. According to statistical analysis, *f*-ratio = 91.5, *p* < 0.00001 (number of repetitions *n* = 3).

Figure 8 demonstrates the results of Shore A hardness measurements for the obtained composite coatings. Previously, a similar study was conducted for the same coatings, but applied to PLA plates [30].

A clear effect of HA addition on hardness was observed, as the value clearly increases with the higher proportion of HA in the materials. For the coatings without the ceramic phase, i.e., **A** and **B**, similar relatively low results were obtained. However, the influence of HA on hardness is evident, as this value clearly rises with an increase in the content of HA, reaching the highest value of 11.6 for sample **D**. Comparing the results obtained with those previously published for coatings deposited on PLA plates, it can be concluded that the coating itself does not significantly affect this parameter, and the hardness is determined by the substrate to which they are deposited (in this case, PLA).

### 3.4. Static Tensile Test

**Figure 9:**
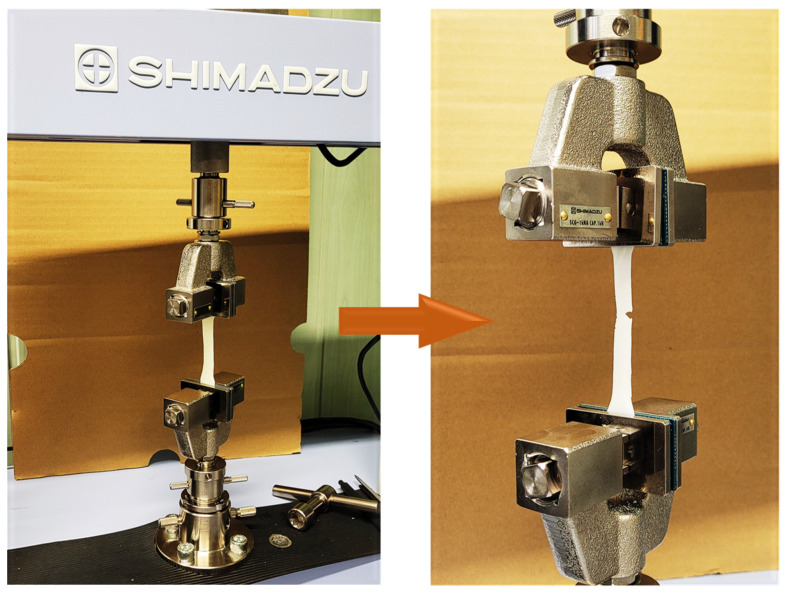
Coating sample during static tensile strength test measurement.

As a result of the study, a clear effect of the ceramic phase and thus the composition of the coatings on the parameters studied was observed. The obtained results are presented in Table 8. The addition of the ceramic phase caused an increase in the modulus of elasticity. Figure 9 demonstrates the process of preparing a sample for measurement.

Comparing polymer samples **A** and **B** with sample **D** containing 15% ceramic phase, an almost ninefold increase in modulus values was observed. At the same time, a decrease in deformation values was observed in the presence of HA. This parameter was almost ten times lower for composite sample **D** compared to polymer sample **A**. Maximum deformation can be defined as the tendency of a material to change shape or deform when a tensile force is applied. Deformation occurs when, as a result of stretching an elastic material, internal intermolecular forces oppose the applied force [42]. Thus, it can be concluded that the presence of HA significantly affects the strength parameters of the developed coatings. No significant effect of collagen addition was observed.

## 4. Discussion

Polymer-based composite materials with a suspended ceramic phase are well-known and extensively studied materials in skeletal regeneration applications. However, their potential for use as coating materials represents a niche. The key is the selection of components to obtain a fully crosslinked and homogeneous material. This study presents a method for obtaining coating materials. These were subjected to in vitro incubation tests in order to determine their behavior under conditions similar to those in a living organism. The mechanical parameters of the materials were also determined. The incubation studies were conducted in four incubation fluids—SBF, artificial saliva, Ringer’s fluid, and distilled water—in order to check the behavior of the coatings in environments with slightly different environmental conditions. Changes in pH value, ionic conductivity, and sorption capacity were examined. Some correlations between the first two parameters were shown. In artificial saliva where the greatest changes in pH were observed, there was the greatest increase in ionic conductivity values. Moreover, the greatest degree of degradation was also observed in this fluid. The materials demonstrated the greatest pH stability in Ringer’s fluid and SBF, and in these fluids, the ionic conductivity was also similar. In all cases, the conductivity values were higher for composite coatings than for polymeric ones. The effect of the proportion of the ceramic phase on the sorption capacity was also observed. The higher the proportion of the ceramic phase, the lower the swelling capacity of the biomaterials. The swelling results were compared to a similar work dealing with the development of injectable hydrogels for hard tissue. Although the purpose was different, the chemical composition of the material was similar, consisting of PEG, nanometer-sized ceramics, and PEGDA. In this case, a significantly higher increase in swelling capacity was observed [43]. However, the cited work used PEG with a molecular weight of 35,000 g/mol, while for the coatings, PEG with a molecular weight of 10,000 g/mol was used. It can therefore be speculated that molecular weight may affect the sorption capacity. Unfortunately, the exact weight for PEGDA was not given. Significantly, the changes in conductivity values confirm the existence of interactions between the incubation fluids and the coatings, as confirmed by SEM and EDS analysis. The formation of new apatite crystals and probably chloride crystals was detected. Once again, a correlation due to the presence of HA in the material was demonstrated, as no new apatite precipitates were observed in coatings where it was absent. On the other hand, as little as 5% of HA was enough for the entire surface to be covered with new apatite layers within 14 days, which is a very desirable phenomenon, indicating the ability of the coatings to undergo biomineralization, which is needed for the target application of the developed materials: coating metallic and polymeric implants and ensuring their bioactive nature. Observations of surface changes and the biomineralization process are consistent with reports in the literature. For composites based on PVP and nano-HA, new apatite crystals were already observed after incubation in SBF at 3 and 7 days [44]. However, the origin of the hydroxyapatite should also be considered in this type of application, as its bioactivity can vary depending on whether it is natural or synthetic [45].

The analysis of mechanical properties demonstrated that the materials did not have a high modulus of elasticity despite the presence of HA, which has high strength parameters. This is probably because the coatings were relatively thin (the thickness of the coatings was previously reported in [30]). However, in this case, its influence on the measured parameter can also be clearly seen, with the modulus of elasticity being higher with more ceramics in the polymer phase. A similar relationship was observed for the measurement of hardness; relating this to previous studies, it can be concluded that once the coating is applied to the target implant/biomaterial, the mechanical strength of the entire system will not be determined by the presence of the developed coating, but by the properties of the covered material. The effect of hydroxyapatite reinforcement on polymeric and hydrogel materials has been reported in the literature, where it has been confirmed that it increases the modulus of elasticity. In other composites based on PEG or PVP and polyvinyl alcohol, the value of modulus of elasticity was up to four times higher, and the final material was able to carry greater loads [43,46].

Nevertheless, with reference to the discussion presented above, it should be emphasized that the factors analyzed in the presented manuscript, mainly mechanical resistance and incubation fluids, are not the only ones that affect the implanted biomaterial in the body. Biological fluids, besides the considered ions, contain various types of proteins and other active components that can affect the implanted material via surface energy changes, hydrophilic/hydrophobic interactions, and charge changes through cationic/anionic binding [47].

## 5. Conclusions

This work describes the physicochemical and mechanical analysis of composite coatings intended for coating implants for craniofacial bone regeneration. The stability of the materials was demonstrated in incubating fluids such as SBF, with no drastic changes in pH value or material degradation observed. Moreover, sorption capacity analysis showed that the materials are able to bind fluids and swell, which enables their potential use as carriers of active substances in targeted therapy. The coatings themselves do not have high tensile strength or hardness, and this parameter is determined by the substrate that is covered with them. Analyzing the results obtained, it can be concluded that the application of coatings on an implantable material will not change its mechanical properties, but will add the desired bioactivity. This parameter is determined by the nature of HA with its ability to osteoconduct and precipitate new apatite layers, and was confirmed by SEM and EDS analysis. The results obtained confirm the need for further research on the developed composite coatings, including cytotoxicity studies to eliminate possible negative effects on tissues.

## 6. Patents

The results of extensive research, part of which is presented in this manuscript, are three patent applications to the Polish Patent Office: no: P.442978, application date: 29 November 2022; no: P.442979, application date: 29 November 2022; no: P.442980, application date: 29 November 2022.

## Figures and Tables

**Table 1:** Coating composition.

Sample Symbol	PVP (mL)	PEG (mL)	GSH (g)	COL (g)	HA (% *w*/*v*)	PEGDA (mL)	Photoinitiator (µL)
**A**	5	5	2	-	-	1.8	50
**B**				0.04	-		
**C**					5		
**D**					15		

**Table 2:** Reagents for preparation of artificial saliva (pH 5.5, 1 L).

Order	Reagent	Amount (g/1000 mL)
#1	NaCl	0.400
#2	KCl	0.400
#3	CaCl_2_·2H_2_O	0.795
#4	Na_2_HPO_4_·H_2_O	0.780
#5	Na_2_S·9H_2_O	0.005
#6	CO(NH_2_)_2_	1.000

**Table 3:** Reagents for preparation of SBF (pH 7.4–7.45, 1 L).

Order	Reagent	Amount (g/1000 mL)
#1	NaCl	8.035
#2	NaHCO_3_	0.355
#3	KCl	0.225
#4	K_2_HPO_4_·3H_2_O	0.231
#5	MgCl_2_·6H_2_O	0.311
#6	1 M HCl	40 mL
#7	CaCl_2_	0.292
#8	Na_2_SO_4_	0.072
#9	Tris	6.118
#10	1 M HCl	Appropriate amount for adjusting pH

**Table 4:** Reagents for preparation of Ringer’s solution (pH 6.4–6.5, 1 L).

Order	Reagent	Amount (g/1000 mL)
#1	NaCl	8.600
#2	KCl	0.300
#3	CaCl_2_·2H_2_O	0.480

**Table 5:** Rate parameter (τ) and equilibrium swelling (Se) of tested samples in SBF, Ringer’s fluid, artificial saliva, and water.

Sample Type	SBF		Ringer’s Fluid		Artificial Saliva		Water	
	Se (%)	τ	Se (%)	τ	Se (%)	τ	Se (%)	τ
**A**	109.67 ± 4.49	23.65 ± 5.81	107.01 ± 4.44	34.45 ± 5.83	104.91 ± 4.17	23.00 ± 5.45	115.8 ± 5.00	30.41 ± 8.11
**B**	114.27 ± 4.93	20.79 ± 5.44	111.77 ± 4.81	21.68 ± 5.64	109.73 ± 4.65	21.17 ± 5.42	118.8 ± 5.13	29.70 ± 8.04
**C**	99.04 ± 4.23	30.01 ± 7.69	96.81 ± 4.02	30.86 ± 7.67	95.14 ± 3.86	31.14 ± 7.53	103.81 ± 4.52	51.14 ± 10.91
**D**	80.11 ± 1.52	54.16 ± 10.17	77.11 ± 1.48	51.88 ± 9.77	73.68 ± 0.86	46.84 ± 5.39	82.51 ± 2.79	47.30 ± 9.22

**Table 6:** Degradation of materials during incubation indicated by mass difference (%).

Sample Type	Mass Difference (%)			
	SBF	Ringer’s Fluid	Artificial Saliva	Distilled Water
**A**	12.12	22.93	36.19	16.30
**B**	12.46	23.65	37.41	15.58
**C**	10.84	20.85	37.89	15.56
**D**	8.14	20.25	39.79	14.18

**Table 7:** Elemental composition of tested coatings after incubation in SBF for each EDS point.

Sample Type	Atomic Percentage (%)
**A**	C: 21.08, O: 54.13, Na: 3.61 Mg: 0.18, P: 0.40, Cl: 4.51, K: 0.12, Ca: 15.99 C: 67.64, O: 22.49, Na: 3.52, Mg: 0.28, P: 0.92, Cl: 4.47, K: 0.26, Ca: 0.43
**B**	C: 52.94, O: 25.89, Na: 2.05 Mg: 0.32, P: 0.90, Cl: 5.53, K: 0.40, Ca: 11.97 C: 69.37, O: 18.79, Na: 2.31, Mg: 0.14, P: 2.64, Cl: 5.49, K: 0.39, Ca: 0.88
**C**	C: 34.78, O: 33.51, Na: 3.08, Mg: 0.49, P: 10.84, Cl: 3.82, K: 0.10, Ca: 13.40 C: 33.93, O: 34.20, Na: 2.57, Mg: 0.29, P: 7.93, Cl: 2.14, K: 0.10, Ca: 10.85
**D**	C: 38.52, O: 32.41, Na: 0.77, Mg: 0.13, P: 8.94, Cl: 1.09, K: 0.13, Ca: 18.02 C: 36.92, O: 38.72, Na: 1.09, Mg: 0.33, P: 8.06, Cl: 0.93, K: 0.06, Ca: 13.89

**Table 8:** Young’s modulus and maximum deformation parameters of the obtained materials (number of repetitions *n* = 3).

Sample	Modulus of Elasticity (N/mm^2^)	Max. Deformation (%)
**A**	11.48 ± 0.61	20.51 ± 1.78
**B**	13.22 ± 3.46	15.71 ± 0.38
**C**	48.22 ± 3.87	4.63 ± 0.48
**D**	93.31 ± 11.18	2.15 ± 1.01

## Data Availability

The data that support the findings of this study are contained within the article.
